# An exploratory case series of sequential stereotactic body radiotherapy and tislelizumab after platinum-doublet chemotherapy in stage IIIB/C–IV non-squamous NSCLC

**DOI:** 10.3389/fimmu.2026.1854773

**Published:** 2026-09-07

**Authors:** Shanshan Li, Feng Du, Changjun Leng, Jianjian Dou, Jian Shi, Yan Zhang, Feifei Lu, Qiang Wang

**Affiliations:** Department of Oncology, Zibo Municipal Hospital, Zibo, Shandong, China

**Keywords:** efficacy, immune checkpoint inhibitor, non-small cell lung cancer, safety, stereotactic body radiotherapy

## Abstract

**Background:**

Patients with unresectable stage IIIB/C-IV non-squamous non-small cell lung cancer (NSCLC) lacking driver mutations (EGFR/ALK/ROS1/RET/BRAF/MET) face limited treatment options. This single-center prospective exploratory pilot case series, limited to a small pre-selected cohort, aims to descriptively characterize real-world efficacy, survival signals and safety profiles of sequential platinum-doublet chemotherapy followed by individualized stereotactic body radiotherapy (SBRT) combined with tislelizumab; this work cannot draw definitive therapeutic conclusions and only generates preliminary hypothesis-building observations.

**Methods:**

This exploratory pilot case series enrolled only patients who maintained disease control after 4-cycle platinum-based chemotherapy (introducing notable selection bias favoring chemo-sensitive tumors). Individualized SBRT with heterogeneous dose/fractionation schemes (3–10 Gy/fx) was delivered to metastatic lesions, with tislelizumab 200 mg q3w initiated within five SBRT fractions and maintained until progression or intolerable toxicity. We added standardized multi-modal toxicity surveillance and grading-based intervention protocols for differentiating radiation versus immune pneumonitis. Endpoints were analyzed purely for descriptive purposes, including 1-year PFS rate, median PFS, OS, ORR, DCR, and treatment-related adverse events (TRAEs).

**Results:**

The 1-year PFS rate was 25%. The median PFS was 10.14 months (95%CI: 3.65–17.38). Median OS was 26.89 months (95%CI: 17.68–30.19), with 1-year and 2-year OS rates of 100% and 62.5%, respectively. The best overall response rate (BOR), defined as the best response recorded from the start of treatment until disease progression or initiation of new anticancer therapy, was 87.5%, with 7 patients achieving partial response (PR) and 1 patient achieving stable disease (SD) as best response. At the fixed 1-year time point, the ORR was 12.5% (1/8 patients in PR) and the DCR was 100% (1 patient in PR and 7 patients in SD). Treatment-related adverse events (TRAEs) were observed in all patients (100%). The incidence of grade ≥3 TRAEs was 62.5%.

**Conclusion:**

This small exploratory case series provides only preliminary, hypothesis-generating signals regarding the feasibility of sequential SBRT and tislelizumab after chemotherapy in selected patients with advanced nsNSCLC. The observed efficacy and safety data are insufficient to establish definitive conclusions or clinical recommendations. These findings require rigorous validation in larger, controlled trials before any therapeutic inference can be drawn.

## Introduction

Lung cancer remains the leading cause of cancer-related deaths worldwide, severely affecting patient health and posing a substantial economic burden on society (1), especially in advanced stages, where non-small cell lung cancer (NSCLC) accounts for approximately 85% of cases (2). For patients with unresectable stage IIIB/C-IV non-squamous NSCLC (nsNSCLC) lacking actionable driver mutations (EGFR/ALK/ROS1/RET/BRAF/MET), treatment options are limited and new treatment approaches need to be explored to improve patient outcomes.

Immunotherapy, particularly programmed cell death protein 1 (PD-1) or programmed death-ligand 1 (PD-L1) inhibitors, has revolutionized the management of advanced NSCLC lacking actionable driver mutations (3). Tislelizumab, a humanized IgG4 anti-PD-1 monoclonal antibody, has shown significant efficacy in multiple clinical trials, including the RATIONALE 304 and 307 studies, leading to its approval in China for advanced NSCLC (4, 5);. However, only a subset of patients responds to immune checkpoint inhibitors alone, highlighting the need for combination strategies to enhance antitumor immunity.

Radiotherapy, especially stereotactic body radiotherapy (SBRT), can induce immunogenic cell death, enhance tumor antigen presentation, and promote T-cell infiltration, potentially synergizing with immunotherapy (6). The PACIFIC trial established consolidation immunotherapy after chemoradiotherapy as standard of care in stage III NSCLC, but the role of sequential radiotherapy combined with immunotherapy in stage IV disease remains underexplored (7).

Notably, this investigation is designed as a small single-center exploratory pilot case series rather than a definitive randomized or large-cohort therapeutic trial. Its primary objective is to descriptively document real-world treatment trajectories, response signals and toxicity management experience for sequential chemo-SBRT plus tislelizumab, instead of validating the superiority or definite clinical efficacy of this multimodal regimen. Upfront limitations including limited sample size, selection bias from post-chemotherapy enrollment, and individualized heterogeneous SBRT plans are acknowledged to restrict generalizable clinical inference from our observations.

We conducted this single-center exploratory pilot case series to descriptively record real-world response, survival and safety profiles of sequential platinum chemotherapy plus individualized SBRT combined with tislelizumab in patients with unresectable stage IIIB/C-IV nsNSCLC without driver mutations.

## Methods

### Study design and participants

This was a prospective, single-arm, observational study conducted at Zibo Municipal Hospital. The initial study design planned to enroll 20 patients based on feasibility and expected recruitment rates. Due to slower-than-anticipated enrollment during the COVID-19 pandemic and subsequent changes in clinical practice, we present an interim analysis of the first 8 patients with a minimum follow-up of 24 months. Eligible patients were (1): aged 18–75 years (2); with histologically or cytologically confirmed, unresectable or recurrent/metastatic stage IIIB/C-IV nsNSLC according to the 8th edition of the AJCC TNM staging system (3); negative for actionable sensitizing alterations in EGFR, ALK, ROS1, RET, BRAF, and MET. Molecular Profiling: all patients underwent comprehensive genomic profiling using next-generation sequencing (NGS) panels (OncoScreen Plus, Burning Rock Dx, China; panel size 500 genes) for EGFR, ALK, ROS1, RET, BRAF, and MET alterations at initial diagnosis. PD-L1 expression was assessed by immunohistochemistry (22C3 antibody, Dako) but was not required for enrollment; Metastatic burden was defined as the number of metastatic organs involved and the total number of metastatic lesions. Oligometastatic status was classified as ≤5 metastatic lesions across ≤3 organs (4). had completed four cycles of platinum-based doublet chemotherapy (pemetrexed plus cisplatin or carboplatin) without evidence of progressive disease (complete response, partial response, or stable disease per RECIST v1.1) (5); Eastern Cooperative Oncology Group Performance Status (ECOG PS) 0-2 (6); adequate hematologic, hepatic, and renal function (7); at least one measurable lesion per RECIST v1.1 (8); life expectancy ≥12 weeks. Key exclusion criteria included (1): mixed small-cell or sarcomatoid histology (2); prior exposure to immune checkpoint inhibitors (3); active or steroid-requiring non-infectious pneumonitis within one year (4); symptomatic central nervous system metastases requiring immediate local therapy (5); active autoimmune disease requiring systemic immunosuppression (6); uncontrolled intercurrent illness including active infection, symptomatic congestive heart failure, unstable angina, or cardiac arrhythmia (7); pregnancy or breastfeeding (8); any condition that would interfere with study compliance. This study was prospectively registered at ClinicalTrials.gov (NCT07198217) and approved by the Medical Ethics Committee of Zibo Municipal Hospital (No. 2021-Institutional Ethics-01).

### Treatment

After chemotherapy, patients received SBRT to residual or metastatic sites (3–10 Gy per fraction, once daily, five fractions per week). Multi-lesion irradiation was permitted with individualized dosing. Tislelizumab (200 mg IV Q3W) was initiated during or within five fractions of SBRT and continued as maintenance until disease progression, unacceptable toxicity, or study withdrawal.

The biologically effective dose (BED) for each irradiated lesion was calculated using the linear-quadratic model with an α/β ratio of 10 Gy for tumor tissue:

BED = n × d × (1 + d/(α/β))

where n is the number of fractions and d is the dose per fraction in Gy. This calculation was applied to all lesions to enable cross-fractionation comparisons.

### Assessments

The primary endpoint was 1-year progression-free survival (PFS) rate. Secondary endpoints included median PFS, overall survival (OS), objective response rate (ORR), disease control rate (DCR), and safety. Best overall response (BOR) was defined as the best response recorded from the start of treatment until disease progression or initiation of new anticancer therapy. Tumor response was assessed by investigators per RECIST v1.1 every 6 weeks for the first 18 weeks, then every 8–12 weeks. Adverse events (AEs) were graded per CTCAE v5.0.

### Toxicity monitoring and standardized intervention protocols

Routine Surveillance Schedule: (1) Baseline examination: contrast-enhanced chest CT, pulmonary function test, complete blood count, inflammatory markers, thyroid function test before combined SBRT-tislelizumab therapy. (2) During SBRT course: weekly inquiry of respiratory symptoms (cough, dyspnea, fever) and routine hematologic laboratory testing. (3) Post-SBRT consolidation immunotherapy: chest CT scan every 6 weeks, inflammatory biomarkers detected per tislelizumab cycle, continuous long-term respiratory symptom follow-up.

Differentiation Algorithm for Radiation Pneumonitis (RP) vs Immune-Related Pneumonitis (irP): RP typically emerges within 3 months after thoracic SBRT, with radiological infiltrates strictly confined to prior radiation fields; irP can occur at any time during anti-PD-1 treatment, presenting as diffuse out-of-field interstitial lesions. All suspected interstitial lung adverse events received multidisciplinary consultation of radiation oncology, medical oncology and pulmonology to distinguish the two entities.

Grading-Based Intervention Criteria for All TRAEs (1): Hematologic toxicity (neutropenia, leukopenia, thrombocytopenia): Grade 1–2 only received supportive hematopoietic care; Grade ≥3 triggered immediate tislelizumab hold, granulocyte/thrombopoietin stimulating factor support, and immunotherapy cycle delay until blood count recovery. No SBRT dose reduction was applied for isolated hematologic toxicity during radiotherapy (2). Cutaneous immune adverse events (including toxic epidermal necrolysis, TEN): Grade 1–2 treated with topical glucocorticoids and antihistamines, tislelizumab maintained; Grade 3 TEN required 1–2 mg/kg/d systemic methylprednisolone, with permanent tislelizumab discontinuation after full skin recovery without re-challenge (3). Pulmonary toxicity (RP/irP): Grade 1: close clinical and imaging observation; Grade 2: oral prednisone 0.5 mg/kg/d; Grade ≥3: intravenous methylprednisolone 1–2 mg/kg/d, immediate suspension of tislelizumab, permanent discontinuation for recurrent grade ≥3 pneumonitis. SBRT fractions were temporarily delayed if grade ≥2 respiratory symptoms developed during radiotherapy.

SBRT Dose Adjustment Rules: If concurrent grade ≥2 non-hematologic toxicity (pulmonary/cutaneous) occurred during fraction delivery, remaining SBRT fractions were delayed until toxicity improved to grade ≤1. Pre-emptive upfront SBRT dose de-escalation was not implemented in this cohort per institutional standard practice.

### Statistical analysis

Efficacy and safety were analyzed in the intention-to-treat population. All survival and response analyses were performed solely for descriptive visualization via the Kaplan–Meier method; no inter-group comparison, subgroup testing, or dose-response correlation analysis was conducted due to the extremely small sample size and heterogeneous SBRT regimens. Median PFS and OS with 95% CIs were only reported as descriptive reference values without statistical inference.

## Results

### Patient characteristics

As of March 25, 2025, 8 patients were enrolled. Baseline characteristics are summarized in **Table 1**. Median age was 60 years (range 52-73), and sex was equally distributed (50% male, 50% female). The majority had an ECOG PS of 1 (75%; none had ECOG PS ≥ 2). Most patients were never-smokers (87.5%), with only one current smoker (12.5%). All patients presented with stage IV disease (50% IVA, 50% IVB) and at least one metastatic site. All patients had histologically confirmed adenocarcinoma. The most common metastatic locations were bone and contralateral lung (each 37.5%), followed by liver (25%) and brain (12.5%). All participants underwent PD-L1 TPS detection: 37.5% (n=3) TPS <1%, 12.5% (n=1) TPS 1–49%, and 50.0% (n=4) TPS ≥50%. Median metastatic organ and lesion counts were 2 (range 1–4) and 3 (range 1–5), respectively; 62.5% (n=5) had oligometastatic disease (≤5 lesions, ≤3 organs). One patient had untreated asymptomatic brain metastases, and 7 patients were free of intracranial lesions.

**Table 1:** Baseline patient characteristics.

Characteristics	
Median age, years(range)	60 (52–73)
Sex, n (%)	
Male	4(50.0)
Female	4(50.0)
ECOG PS, n (%)	
0	2(25.0)
1	6(75.0)
2	0
Smoking history, n (%)	
Former	0
Never	7(87.5)
Current	1(12.5)
Disease stage, n (%)	
IV A	2(25.0)
IV B	6(75.0)
Histology, n (%)	
Adenocarcinoma	8 (100%)
Metastases, n (%)	
Any metastases	8 (100)
Liver metastases	2(25.0)
Brain metastases	1(12.5)
Osseous metastases	3(37.5)
Contralateral lung metastases	3(37.5)
PD-L1 expression, n (%)	
TPS <1%	3 (37.5)
TPS 1–49%	1 (12.5)
TPS ≥50%	4 (50.0)
Metastatic burden	
Number of metastatic organs, median (range)	2 (1–4)
Number of metastatic lesions, median (range)	3 (1–5)
Oligometastatic disease (≤5 lesions, ≤3 organs)	5 (62.5)
Brain metastases management	
Asymptomatic, no immediate therapy	1 (12.5)
None	7 (87.5)

ECOG PS, Eastern Cooperative Oncology Group Performance Status.

Details of metastatic sites, lesions treated with SBRT, and immunotherapy for each patient are presented in **Supplementary Table 1**. As shown in **Supplementary Table 1**, lesion selection for SBRT was individualized; not all metastatic sites received radiotherapy, lesion selection was done by the investigator on a patient-by-patient basis, and Tislelizumab was added at Week 1 of radiotherapy initiation.

### Efficacy

**Figure 1:**
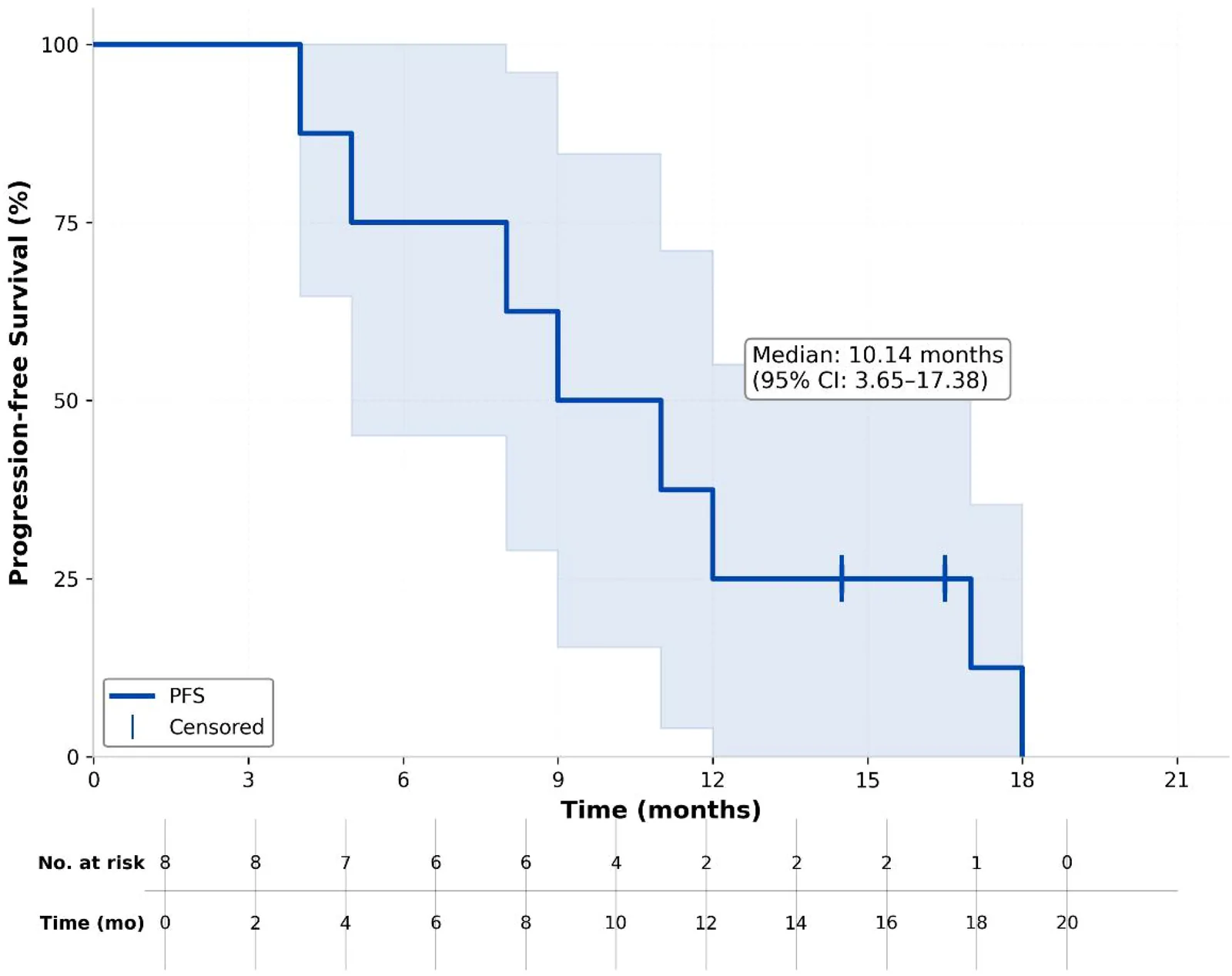
Kaplan-Meier curves of PFS. Tick marks indicate censored observations. The table below the graph shows the number of patients at risk at the corresponding time points. Median PFS was 10.14 months (95% CI: 3.65–17.38). The shaded area represents the 95% CI.

**Figure 2:**
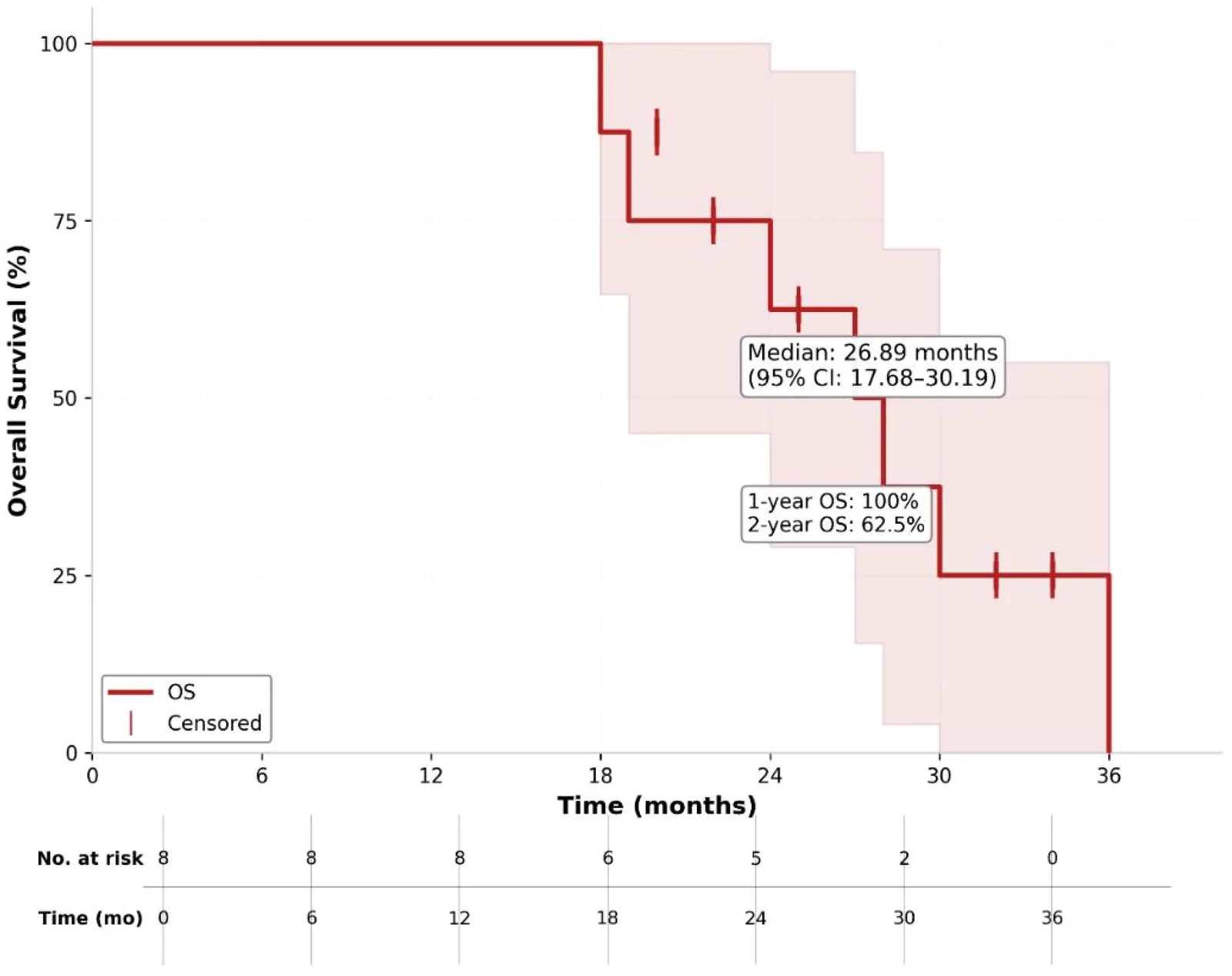
Kaplan-Meier curves of OS. Tick marks indicate censored observations. The table below the graph shows the number of patients at risk at the corresponding time points. Median OS was 26.89 months (95% CI: 17.68–30.19). The 1-year and 2-year OS rates were 100% and 62.5%, respectively. The shaded area represents the 95% CI.

The median follow-up time was 25.6 months, and the results showed that the best overall response rate (BOR) was 87.5% (**Table 2**), assessed according to RECIST v1.1. **Table 2** shows that 4 patients achieved partial response (PR) during chemotherapy and maintained PR during SBRT+ immunotherapy. 4 patients achieved stable disease (SD) during chemotherapy, 3 of whom further improved to PR during SBRT+ immunotherapy. **Table 3** shows that at the fixed 1-year time point, 1 patient (12.5%) remained in PR, 7 patients had SD (87.5%), yielding a 1-year DCR of 100%. The 1-year PFS rate was 25.0%, the median PFS was 10.14 months (95% CI: 3.65-17.38; **Figure 1**), ORR was 12.5% (**Table 3**). Median OS was 26.89 months (95% CI: 17.68-30.19; **Figure 2**), with 1-year and 2-year OS rates of 100% and 62.5%, respectively.

**Table 2:** Best overall response (from pre-chemotherapy baseline).

Patient	Response to chemotherapy	Response to SBRT+Tislelizumab (from pre-chemo)
1	PR (-26%)	PR (-52%)
2	SD (-18%)	PR (-62%)
3	PR (-30%)	PR (-62%)
4	SD (-11%)	PR (-58%)
5	PR (-32%)	PR (-59%)
6	SD (-3%)	PR (-50%)
7	PR (-34%)	PR (-67%)
8	SD (-2%)	SD (-5%)

PR, Partial Response; SD, Stable Disease; SBRT, stereotactic body radiotherapy.

**Table 3:** Response status of patients at fixed 1-year time.

Efficacy evaluation	1-year
Partial response	1
Stable disease	7
Progressive disease	0
ORR	12.5%
DCR	100%

ORR, objective response rate; DCR, disease control rate.

The BOR of 87.5% represents cumulative response from pre-chemotherapy baseline. Of note, 3 of all 4 SD patients achieved PR as best overall response after chemotherapy, indicating continued tumor shrinkage during the SBRT+ immunotherapy phase.

Notably, prominent inter-patient heterogeneity in SBRT dose, fractionation and irradiated metastatic target selection was observed across this 8-patient cohort. No formal statistical analysis linking radiotherapy BED_10_ or irradiated lesion burden to tumor response, PFS or OS was feasible, and no conclusions regarding optimal SBRT parameters can be drawn from these limited descriptive data.

**Figure 3:**
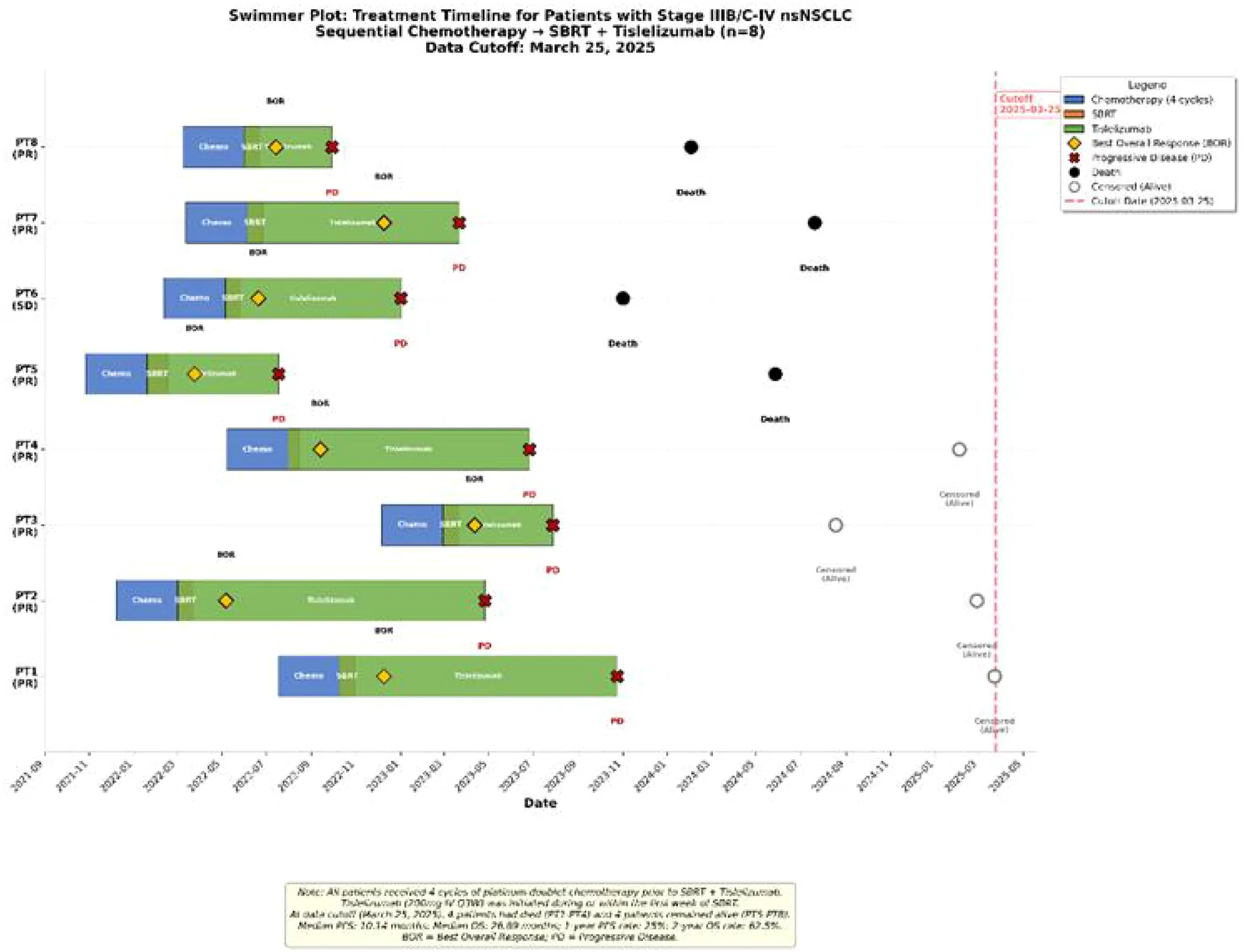
Descriptive swimmer plot displaying individual patient treatment and disease trajectories for the 8-patient exploratory cohort of stage IIIB/C-IV nsNSCLC. This visualization serves only as case-level descriptive documentation rather than formal statistical plotting, given the limited sample size, and is not intended for population-level statistical inference.

**Figure 3** provides descriptive case-by-case visualization of individual patient treatment and disease trajectories; due to the study’s very small sample size, this figure carries no statistical inferential value and only documents each patient’s clinical course separately. All patients completed chemotherapy followed by SBRT and tislelizumab. 3 patients with initial SD subsequently achieved PR, whereas 1 patient remained in SD throughout. At data cutoff, 6 (75%) had progressed, 2 (25%) were progression-free at 1 year, and 4 (50%) were alive. No treatment-related deaths occurred.

### Safety

All patients (100%) experienced treatment-related adverse events (TRAEs), with an incidence of grade ≥3 TRAEs of 62.5%. The most common TRAEs included neutropenia (37.5%), leukopenia (25%), and thrombocytopenia (12.5%). The incidence of immune-related adverse events (irAEs) was 25%, which included leukopenia (12.5%) and toxic epidermal necrolysis (12.5%). No treatment-related deaths occurred (**Table 4**).

**Table 4:** Safety in all patients.

TRAEs	Any Grade n (%)	Grade 1–2 n (%)	≥ Grade 3 n (%)
Any	8 (100%)	8 (100%)	5 (62.5%)
Leukopenia	6 (75%)	4 (50.0%)	2 (25%)
Neutropenia	6 (75%)	3 (37.5%)	3 (37.5%)
Thrombocytopenia	2 (25%)	1 (12.5%)	1 (12.5%)
Red blood cell reduction	3 (37.5%)	3 (37.5%)	0
Hypothyroidism	4 (50%)	4 (50.0%)	0
Elevated α-hydroxybutyrate dehydrogenase	4 (50%)	4 (50.0%)	0
Elevated troponin I	1 (12.5%)	1 (12.5%)	0
Elevated γ-glutamyltransferase	3 (37.5%)	3 (37.5%)	0
Elevated alanine aminotransferase	2 (25%)	1 (12.5%)	1 (12.5%)
Elevated glutamic-oxaloacetic transaminase	2 (25%)	2 (25%)	0
Toxic epidermal necrolysis	1 (12.5%)	0	1 (12.5%)
Nausea	1 (12.5%)	0	1 (12.5%)
Vomiting	1 (12.5%)	0	1 (12.5%)
Elevated creatinine	2 (25.0%)	2 (25.0%)	0

One patient developed grade 3 toxic epidermal necrolysis 2 cycles after tislelizumab initiation. Systemic methylprednisolone 1.5 mg/kg/d was administered for 4 weeks with gradual tapering, combined with full-thickness skin wound supportive care. Tislelizumab was permanently discontinued, and the patient achieved complete cutaneous recovery without residual organ sequelae after 3 months of follow-up.

## Discussion

Non-squamous NSCLC is a prevalent and deadly form of lung cancer that significantly impacts patient health and incurs substantial economic burdens, particularly in advanced stages (8). Current treatment modalities, including chemotherapy, targeted therapies, and immunotherapies, often exhibit limitations in efficacy and tolerability, necessitating the exploration of novel therapeutic strategies to enhance patient outcomes (9). The pressing need for effective interventions is underscored by the high mortality rates associated with this disease, especially among patients who do not harbor sensitive driver gene mutations, which complicate treatment options.

This prospective observational case series was designed as an exploratory pilot investigation to assess the feasibility and preliminary signals of sequential SBRT combined with tislelizumab after chemotherapy in patients with advanced nsNSCLC. We explicitly acknowledge that the small cohort size (n=8), single-arm design, and lack of a comparator group render this study hypothesis-generating only and unsuitable for definitive efficacy claims.

This prospective real-world study suggests preliminary signals that sequential chemotherapy, SBRT, and tislelizumab confer preliminary descriptive anti-tumor signals in patients with advanced nsNSCLC without driver mutations. The descriptive median PFS of 10.14 months and 25% 1-year PFS rate only reflect outcome trends within this pre-selected chemo-sensitive subgroup. The prolonged median OS (26.89 months) and high BOR (87.5%) are partially driven by our strict enrollment filter requiring post-chemotherapy disease control, which enriches patients with inherently favorable tumor biology and prognosis (10–13);. The high ORR of 87.5% and 18-week DCR of 80% underscore the observed numerical response trends of this multimodal approach (14–18);. These response rates exceed those typically reported with immune checkpoint inhibitor monotherapy or chemoimmunotherapy in unselected NSCLC cohorts, only hints at a hypothetical trend of potential synergy; this tentative observation requires rigorous validation in large controlled cohorts. Radiation therapy may enhance the response to anti-PD-1 therapy by increasing tumor antigen release and enhancing tumor cell invasion of CD8-positive T cells (19). The median OS observed in this selected cohort was 26.89 months and the 2-year OS rate was 62.5%. Although cross-trial comparisons are limited by study design and patient population, the descriptive survival trend in this chemo-selected subgroup observed in this study is encouraging considering previously reported Phase III trials of first-line chemoimmunotherapy in NSCLC populations. KEYNOTE-189 reported a median OS of 22.0 months and a 2-year OS rate of 45.7% with pembrolizumab plus chemotherapy in the ITT population (11). The TELMA trial reported a 2-year OS rate of 44.7% with atezolizumab plus bevacizumab in high-TMB nsNSCLC (20). CheckMate 9LA study results showed that the median PFS for first-line immunotherapy in metastatic NSCLC was 15.8 months (21). Cross-trial historical comparisons are only provided as loose descriptive reference and cannot be interpreted as evidence of independent anti-tumor superiority from the SBRT plus tislelizumab combination; random error from the n=8 cohort further limits reliable comparison against external datasets.

The safety profile demonstrated a high incidence of TRAEs (100%) and grade ≥3 events (62.5%), predominantly hematologic toxicities likely attributable to cumulative platinum-based chemotherapy. Immune-related adverse events occurred in 25% of patients, including one case of toxic epidermal necrolysis, underscoring the need for vigilant monitoring. Standardized grade-based steroids and supportive interventions achieved reversible toxicity remission in all patients with no treatment-related deaths, indicating a manageable toxicity risk under strict close monitoring. Importantly, no cases of grade ≥2 pneumonitis were observed in this series. However, the small sample size and relatively short follow-up preclude confident conclusions about the true incidence of this potentially life-threatening complication. The predefined safety management protocol described herein was implemented prospectively to ensure standardized, reproducible toxicity surveillance and intervention, which we believe is essential for any future studies investigating this combination. Overall, the safety profile of the combination regimen was manageable, with no new safety signals identified, only suggests that toxicity may be manageable under strict, intensive monitoring protocols within this limited small cohort; generalizable safety conclusions cannot be drawn (22–24);.

Thoracic SBRT combined with PD-1 blockade confers overlapping pulmonary toxicity risk, which necessitates standardized structured surveillance to differentiate radiation pneumonitis and immune-related pneumonitis, two entities with divergent therapeutic strategies and long-term prognosis. In this case series, we implemented the multi-modal toxicity monitoring and intervention algorithm supplemented in Methods to screen early respiratory adverse events, with all suspected interstitial lung lesions managed via multidisciplinary joint evaluation. Although 62.5% of patients experienced grade ≥3 TRAEs, standardized grading-based steroid and supportive interventions achieved reversible toxicity resolution in all patients without treatment-related death, demonstrating manageable toxicity risk under rigorous close monitoring.

This study is limited by its small sample size, single-arm design, absence of a control group, heterogeneous SBRT protocols, lack of centralized radiologic review, and incomplete biomarker profiling (e.g., tumor mutational burden). In addition, the design introduces selection bias by enrolling only patients without progression after four cycles of chemotherapy—a population with inherently favorable disease biology. The observed PFS (10.14 months) and OS (26.89 months) likely reflect this pre-selection advantage rather than treatment efficacy alone, as chemotherapy-responsive patients historically represent 50-60% of metastatic NSCLC with better outcomes than ITT populations (25). The results should be considered preliminary and are not generalizable. The study was originally planned as a pilot cohort with a target enrollment of 20 patients; slower-than-anticipated accrual during the COVID-19 pandemic and evolving clinical practice patterns resulted in this interim case series analysis. Future large-scale prospective controlled cohorts with unified SBRT dose-fractionation protocols are required to systematically explore optimal BED thresholds, metastatic target selection strategies, and timing windows for tislelizumab integration with thoracic radiotherapy. Controlled study designs are necessary to eliminate the chemotherapy-induced selection bias present in this pilot case series and validate whether the preliminary descriptive survival and response signals observed here translate to consistent clinical benefit in unselected driver-negative advanced nsNSCLC populations.

## Conclusion

In conclusion, this exploratory case series provides preliminary, hypothesis-generating data on the feasibility of sequential SBRT and tislelizumab after platinum-doublet chemotherapy in a highly selected cohort of patients with advanced non-squamous NSCLC without driver mutations. While the observed signals are suggestive of activity, the very small sample size, single-arm design, and selection bias preclude any definitive conclusions regarding efficacy or the establishment of a new therapeutic standard. The safety profile, although manageable with proactive monitoring, carries a substantial risk of severe toxicity that mandates rigorous safety protocols. These findings warrant validation in larger, randomized, controlled trials with appropriate comparator arms, standardized radiotherapy parameters, and comprehensive biomarker analysis before any clinical recommendation can be made.

## Data Availability

The raw data supporting the conclusions of this article will be made available by the authors, without undue reservation.
