# Supplementary material for: An exploratory case series of sequential stereotactic body radiotherapy and tislelizumab after platinum-doublet chemotherapy in stage IIIB/C–IV non-squamous NSCLC
Source: Front Immunol. 2026 Sep 7;17:1854773. doi: 10.3389/fimmu.2026.1854773 (PMC13590561; doi:10.3389/fimmu.2026.1854773)
Supplement: Supplementary file 1 [file Table1.docx]

Supplementary Table S1. Radiation Treatment Details and Immunotherapy Integration.

| Patient | metastatic sites | Treated metastatic sites | SBRT dose (Gy/fraction × fractions) | BED₁₀ | Tislelizumab timing | |
| --- | --- | --- | --- | --- | --- | --- |
| 1 | Hilar and mediastinal lymph nodes, pleura | Lung tumor, hilar and mediastinal lymph nodes | ITV: 4.6 Gy × 15 f, PITV: 4.0 Gy × 15 f | BED_ITV_: 100.7 Gy, BED_PITV_: 84.0 Gy | week 1 | |
| 2 | Contralateral lung, pleura, left ilium | Lung tumor; left ilium | Lung: ITV 4.6 Gy × 15 f, PITV 4.0 Gy × 15f;  Ilium: PTV 3.0 Gy × 10 f | BED_Lung_ _ITV_: 100.7 Gy, BED_Lung_ _PITV_: 84.0 Gy;  BED_Ilium_: 39.0 Gy | week 1 | |
| 3 | Hilar lymph node; liver, brain, right T5 vertebra | Lung tumor; hilar lymph node; liver; right T5 vertebra | Lung: ITV 4.6 Gy × 15 f, PITV 4.0 Gy × 15 f; Hilar LN: ITV 3.5 Gy × 15 f, PITV 3.0 Gy × 15 f; Liver: ITV 4.6 Gy × 15 f, PITV 4.0 Gy × 15 f; T5: PTV 3.0 Gy × 10 f | BED_Lung ITV_: 100.7 Gy, BED_Lung PITV_: 84.0 Gy;  BED_Hilar LN ITV_: 70.9 Gy, BED_Hilar LN PITV_: 58.5 Gy;  BED_Liver ITV_: 100.7 Gy, BE _Liver PITV_: 84.0 Gy;  BED_T5 PTV_: 39.0 Gy | week 1 |  |
| 4 | Hilar lymph node; cervical lymph node; contralateral lung | Hilar lymph node; cervical lymph node | Hilar LN: PTV 3.0 Gy × 20 f;  Cervical LN: PTV 3.0 Gy × 20 f; | BED_Hilar LN PTV_:78.0 Gy;  BED_Cervical LN PTV_:78.0 Gy | week 1 | |
| 5 | Mediastinal lymph nodes, adrenal gland | Lung tumor, mediastinal lymph nodes | ITV 5.0 Gy ×11f, PITV 4.0 Gy × 11 f | BED_ITV_:  82.5 Gy, BED_PITV_: 61.6 Gy | week 1 | |
| 6 | Mediastinal lymph nodes, contralateral lung | Lung tumor, mediastinal lymph nodes | ITV 4.6Gy × 15 f, PITV 4.0 Gy × 15 f | BED_ITV_: 100.7 Gy, BED_PITV_: 84.0 Gy; | week 1 | |
| 7 | Lung, mediastinum and hilar lymph nodes, right ilium | Lung tumor; right ilium | Lung: ITV 4.0 Gy × 15 f, PITV 3.0 Gy × 15 f ;  Right ilium: PTV 3.0 Gy × 20 f | BED_Lung ITV_: 84.0 Gy, BED_Lung PITV_:  58.5 Gy;  BED_Right ilium PTV_ 78.0 Gy | week 1 | |
| 8 | Liver, adrenal gland | Lung; liver | Lung: ITV 4.6 Gy × 15 f, PITV 4.0 Gy × 15 f;  Liver: ITV 4.6 Gy × 15 f, PITV 4.0 Gy × 15 f; | BED_Lung ITV_: 100.7 Gy, BED_Lung PITV_: 84.0 Gy;  BED_Liver ITV_: 100.7 Gy, BED_Liver PITV_: 84.0 Gy; | week 1 | |

ITV: internal target volume; PITV: planning internal target volume; PTV: planning target volume; f: fractions; BED₁₀ = biologically effective dose with α/β = 10 Gy; LN = lymph node; Tislelizumab timing refers to the week of SBRT course when the first dose was administered.
